# The effect of dance mat exergaming systems on physical activity and health – related outcomes in secondary schools: results from a natural experiment

**DOI:** 10.1186/1471-2458-14-951

**Published:** 2014-09-12

**Authors:** Liane B Azevedo, Duika Burges Watson, Catherine Haighton, Jean Adams

**Affiliations:** Health and Social Care Institute, Parkside West, Teesside University, Middlesbrough, TS1 3BA UK; School of Medicine, Pharmacy and Health, Durham University, Stockton-on-Tees, UK; Institute of Health & Society, Newcastle University, Newcastle upon Tyne, UK

**Keywords:** Physical activity, Children, Intervention, Body composition, Health promotion

## Abstract

**Background:**

Exergaming has been proposed as an innovative method for physical activity promotion. However, large effectiveness studies are rare. In January 2011, dance mat systems were introduced in secondary schools in two districts in England with the aim of promoting an innovative opportunity for physical activity. The aim of this natural experiment was to examine the effect of introducing the dance mat exergaming systems on physical activity and health-related outcomes in 11–13 year old students using a non-randomised controlled design and mixed methods.

**Methods:**

Participants were recruited from five schools in intervention districts (n = 280) and two schools in neighbouring control districts (n = 217). Data on physical activity (accelerometer), anthropometrics (weight, BMI and percentage of body fat), aerobic fitness (20-m multistage shuttle run test), health-related quality of life (Kidscreen questionnaire), self-efficacy (children’s physical activity self-efficacy survey), school attendance, focus groups with children and interviews with teachers were collected at baseline and approximately 12 months follow-up.

**Results:**

There was a negative intervention effect on total physical activity (-65.4 cpm CI: -12.6 to -4.7), and light and sedentary physical activity when represented as a percentage of wear time (Light: -2.3% CI: -4.5 to 0.2; Sedentary: 3.3% CI: 0.7 to 5.9). However, compliance with accelerometers at follow-up was poor. There was a significant positive intervention effect on weight (-1.7 kg, 95% CI: -2.9 to -0.4), BMI (-0.9 kg/m^2^, 95% CI: -1.3 to -0.4) and percentage of body fat (-2.2%, 95% CI: -4.2 to -0.2). There was also evidence of improvement in some health-related quality of life parameters: psychological well-being (2.5, 95% CI: 0.1 to 4.8) and autonomy and parent relation (4.2, 95% CI: 1.4 to 7.0).

**Conclusions:**

The implementation of a dance mat exergaming scheme was associated with improvement in anthropometric measurements and parameters of health-related quality of life. However, the mechanisms of these benefits are unclear as there was insufficient data from physical activity to draw robust conclusions. Qualitative findings suggest that there was declining support for the initiative over time, meaning that potential benefits may not have been achieved.

## Background

Schools are an important setting for physical activity promotion [1, 2]. Although there are a large number of studies confirming the short term effectiveness of physical activity interventions in schools, the long term effectiveness of these interventions remains uncertain [3].

Exergames are a type of active video game which include dancing, balance boards simulators and virtual sports simulators [4]. Dance mats are a combination of computer game and exercise in which dance steps are projected onto a wall or screen and players follow them on foot-activated floor pads.

A recent systematic review concluded that exergaming activity may provide light to moderate physical activity in children (less than 18 years old) [5]. Another large randomised controlled trial revealed that exergaming was associated with a significant decrease in body mass index (BMI) and percentage of body fat over 6 months in overweight or obese children aged 10 to 14 years [6]. Others have shown an association between exergaming and improvements in academic behaviour and achievement [7]. However, recent systematic reviews have concluded that there is still limited support regarding the long-term effectiveness of exergaming on physical activity [5, 8, 9].

More recently, explanatory trials [10] have investigated the effect of using exergames in physical education classes and during school breaks. These found that exergames might promote positive attitudes to physical activity, and improve self-efficacy for physical activity [11], cardiorespiratory endurance and academic achievement [12].

Based on this evidence some authors have suggested that the absence of exergaming in schools is a “missed opportunity” to introduce young people to another form of physical activity [13] and this could be an option for policy makers [12]. However, we are not aware of any large pragmatic trials exploring the effect of policy implementation on the use of exergame in schools.

In January 2011, dance mat systems were introduced into 22 public secondary schools (age 11 to 18 years old) in two districts of the North East of England. The initiative was designed to promote an innovative and engaging opportunity for physical activity in schools and was facilitated by the local physical education staff working within and between schools. Beyond this, we are not aware of any specific theory underpinning the intervention. All invited schools agreed to receive the mats and signed a service level agreement which included trialling the mats within the curriculum and sharing them with local primary schools. A steering group of primary care, government and school sports leads co-ordinated the use of the mats and provided top-up funding. The dance mat provider delivered initial training courses to school staff and committed to provide on-going technical and practical support for schools. As researchers, we had no input into design or delivery of the intervention.

This initiative provided a unique opportunity to evaluate the effectiveness of this novel complex public health intervention in a real world setting. As far as we are aware, few studies have used natural experimental methods to examine the implementation of a physical activity intervention in community or school settings with children [14–17]. Furthermore, none have used this research design to examine the effect of an exergame intervention in school children.

Likewise, although the effects of using exergames on physical activity and other associated health benefits in children, including body composition, aerobic fitness and self- efficacy for physical activity, have already been explored [5, 6, 9, 11] it is also possible that exergaming in school has a wider range of outcomes. In particular, effects on health-related quality of life and school attendance have not been studied. Exergaming in schools could impact overall health and consequently have positive impacts on health related quality of life and school attendance [18].

A mixed method study was conducted collecting both quantitative outcome data and qualitative process data. The quantitative data is presented here with brief reference to the qualitative process data. The qualitative data will be presented in full elsewhere. The primary aim of this study was to examine the effect of providing dance mats systems in public secondary schools to 11–13 year old children on physical activity over 12 months. Secondary aims were to study the intervention on BMI, body composition, aerobic fitness, health-related quality of life, self-efficacy for physical activity and school attendance.

## Methods

### Ethics statement

Representatives from schools who received the dance mats were invited to attend a presentation which explained the dance mat scheme and the proposed evaluation. They also received written information on the evaluation and were given the opportunity to ask questions. Eligible children attending schools participating in the evaluation received an information pack which contained a letter to their parent or guardian, an information booklet and written informed consent forms for parent or guardian and an assent form for the child. All children had access to the dance mats, but only participants who signed the assent form and returned a completed informed consent form from their parent or guardian were included in the study. The study received ethical approval from the Faculty of Medical Sciences – Newcastle University (application number: 000318/2010).

### Study design

This was a non-randomised controlled trial with a linked qualitative study. The controlled trial made use of a natural experiment in which the implementation and delivery of the intervention was not manipulated by researchers. Instead, dance mat systems (two sets of 16 mats and a single base unit) were provided to each secondary school in two local authority school districts. Apart from the initial 6 weeks when there was a more structured delivery of dance mats into the curriculum, intervention schools had the freedom to use the dance mats in whatever way they wanted. However, the local authority team who supported the implementation of the dance mats suggested that schools consider using them in scheduled physical education classes, during breaks and lunchtimes, and also outside of school hours as part of ‘enrichment’ activities. Due to the nature of this natural experiment it was not possible to randomly assign schools to intervention or control group.

The study was informed by the MRC guidelines on natural experiments to evaluate population health interventions [19], and included clear definitions of target population and sampling criteria, valid and reliable measures of outcomes, comparison with an unexposed group and qualitative assessment. The qualitative component of this study adheres to RATS (Relevance, Appropriateness, Transparency and Soundness) guidelines on qualitative research [20].

While the technology of the dance mat may be evaluated for outcomes such as physical activity, as a public health intervention understanding how and if the dance mats are valuable to the health of populations required knowing more about the implementation of the intervention, how the mats were used, in what settings, by whom and for what purposes. This linked qualitative process evaluation is complementary and contributes to what Petticrew et al. [20] have termed the ‘jigsaw of evidence’.

Observational studies using accelerometry data have shown that a 10 to 20 min increase in MVPA is associated with lower BMI and waist circumference [21–23]. Therefore, it was estimated that a sample size of 218 pupils per study arm would be required to detect a difference of 10 minutes in the change from baseline in average daily minutes of moderate to vigorous physical activity (MVPA) between intervention and control groups with 90% power at p < 0.05. To allow for missing data and loss to follow up, we aimed to recruit 300 students per study arm. The sample size did not take into account clustering, due to the practical difficulty of recruiting the number of schools required - which would have been a minimum of 4 [24] per arm assuming an intra-cluster correlation of 0.012 [25]. Although, we have recruited the number of schools required for the intervention arm, we have not attained this number for the control. The effect of not taking clustering into account is explored in the data analysis session.

### Participants

Data collection took place between September 2010 and March 2012. All twenty-four schools taking part in the dance mat scheme were invited to participate and five agreed to take part. Nine schools in a neighbouring control district unexposed to the dance mats schemes, matched on size, Index of Multiple Deprivation (IMD) and physical education provision with intervention schools, were invited to participate and two agreed.

Two markers of deprivation were included: IMD and eligibility for free school meals (FSM). Index of Multiple Deprivation is a small-area based marker of deprivation based on a range of measures in seven domains (income; employment; health and disability; education, skills, and training; barriers to housing and services; crime; and the living environment) and is the UK government’s preferred marker of deprivation. Small areas are ranked from 1 (most deprived) to 32,482 (least deprived) [26]. In England, children from families in receipt of a number of income-related welfare benefits are entitled to free school meals.

After obtaining written informed consent from Head Teachers, all Year 7 children (aged 11–12 years; the first secondary school year) at study schools were invited to take part in the research. This particular age group was selected as the transition from primary to secondary school is thought to be critical with respect to its impact on behavioural risk factors. This effect is particularly marked in girls whose physical activity can drop 4% a year between the ages of 11 and 13 years [27]. Exclusion criteria were: having a medical reason that prevented participation in the fitness test, as indicated by the Physical Activity Readiness Questionnaire; having a cardiac pacemaker (as they may have been interfered with bioelectrical impedance); and inability to speak or understand English. Participants received a £10 high street voucher at the end of the study after they returned the accelerometer to thank them for their participation.

### Outcome measures

Baseline (September to December 2010) and follow-up (November 2011 to March 2012) measurements took place at school during lesson time. Sample sizes for each outcome variable varied due to child school absence on data collection days. However, data for the primary outcome – physical activity – were collected from all participants.

#### Physical activity

Participants were asked to wear a physical activity monitor (Actigraph GT3X, DynaPort MiniMod (MiniMod)) around the hip during waking hours (except during water-based activities) for seven consecutive days. To detect the intermittent bouts of moderate to vigorous physical activity data were recorded in 10 second epochs [28]. Data were processed with Actilife version 6.5.4 software (Actigraph, LLC, Pensacola, FL). Accelerometers were considered non-worn if there was a period of 20 consecutive minutes with zero accelerometer counts as this is the most common defined ‘non-wear time’ from studies with children and adolescents [29]. Daily wear-time was calculated by subtracting non-worn time from 24 hours. Data were only processed for participants with wear-times of at least 10 hours per day on at least three days. Ten hours is the suggested minimum time required to provide reliable estimates of physical activity [30]. Although less than 3 valid days can also produce reliable data [30], we have opted to use 3 days as this is the minimum criteria suggested by most previous studies with adolescents and children [31, 32]. Data were processed independent of weekend wear as this has been shown not to affect reliability of data [30].

Total physical activity using vertical axis (VA) and vertical magnitude (VM) data were reported in mean counts per minute (CPM; total counts of activity divided by minutes of wear-time for all valid days). There is contradictory evidence supporting [33, 34] or contesting [35, 36] the added benefit of VM over VA. For this reason, total physical activity was reported using these two different measurements. Using the vertical axis data, Evenson cutpoints [37] were applied to estimate physical activity in minutes per day of sedentary time, light physical activity, moderate physical activity, vigorous physical activity and MVPA. These cutpoints are considered the most accurate to estimate time spent at different exercise intensities in children and adolescents from 5 and 15 years old [38].

Data are presented as average duration of sedentary, light, moderate to vigorous and total physical activity per day. To account for difference in wear time between baseline and follow up measures, the data are also presented as a percentage of wear time (e.g. total sedentary minutes/total wear time *100). This is particularly important as non-wear time appears to distort within and between participants data particularly for sedentary behaviour [39].

#### Anthropometric and body fat measurements

Height was measured to the nearest 0.1 cm using a portable stadiometer (Leicester Height Measure, Child Growth Foundation, London, United Kingdom). Weight and body composition measurements (percentage body fat, fat mass (kg), fat free mass (kg), BMI (kg/m^2^) [40]), were measured using an octopolar tactile-electrode impedance meter (InBody 720, Biospace, Seoul, Korea).

The InBody 720 has been compared with dual-energy X-ray absorptiometry (DXA) in healthy children and adolescents (6 to 18 years old) [41]. Although percentage of body fat was not interchangeable with DXA, InBody measured body composition with high precision in this population (limit of agreement on percentage of body fat: -2.2 +/- 6.1%).

#### Self-efficacy for physical activity and health-related quality of life

Self- efficacy for physical activity was measured with the Children’s Physical Activity Self-Efficacy Survey [42]. The questionnaire included eight statements which were rated on a four-point scale (1- Not at all true, 2- Not very true, 3- Quite true, 4- Very true). Scores for individual items were summed to give a single measure with higher scores indicating greater self-efficacy.

Health–related quality of life was measured with the Kidscreen-27. This was developed and validated in a cross-Europe project [43] and includes 27 questions, over five dimensions: physical well-being, psychological well-being, autonomy and parents, social support, and school environment. Each question offered five possible responses according to intensity of attitude (not at all, slightly, moderately, very, extremely) or frequency (never, seldom, quite often, very often, always). All subscale scores are reported as t-values based on Swiss community normative data, with mean of 50 and standard deviation of 10. Higher scores indicate better quality of life.

#### Aerobic fitness

Aerobic fitness was assessed with the 20 metre multistage shuttle run test, administered with the Bleep test CD (How2become Ltd Kent, UK) developed by the British National Coaching Foundation . This version is a modified version of the original protocol described by Leger et al., 1982 [44] and has been validated as a reliable estimate of VO_2_ max [45]. Participants were instructed to run over a distance of 20 metres marked by cones in time with an audible signal. The test started at a running speed of 8.5 km/h and increasing by 0.5 km/h for each level. Researchers noted the final shuttle when participants reached exhaustion or failed twice to keep up with the bleep intervals. The running speed from the final level and the total number of shuttles completed were recorded.

#### Qualitative data

In-depth interviews were undertaken with teachers involved in the intervention at baseline (20 intervention, 8 control) and at 12- month follow-up (12 intervention, 4 control). Focus groups with pupils groups (n = 2-5 in each setting) at baseline and follow-up were undertaken in all participating schools. Participants were asked about use of the mats and barriers and facilitators to use.

### Data analysis

All analyses were conducted using SPSS (version 18). The results are presented as means and standard deviations. Chi-square tests were performed to compare gender splits between groups at baseline. Characteristics of participants (including, body composition, health-related quality of life, self-efficacy, aerobic fitness, physical activity and school attendance) at baseline were compared using independent sample t-tests. Descriptive differences between follow-up and baseline are presented for each outcome measurement.

The design effect was estimated by the ratio of the total number of participants required using cluster randomisation to the number required using individual randomisation [46]. An inter-cluster correlation coefficient (ICC) for MVPA of 0.012 was estimated from the baseline data presented in a previous study [25]. A design effect of 1.58 was found using an average cluster sample size for MVPA of 50 participants at baseline (Design effect = 1 + (m – 1) * ICC, m = number of people in the cluster; Design effect = 1 + (50–1) * 0.012; Design effect = 1.58). Therefore, considering that there were only two clusters from the control group and the design effect was relatively small, data analysis was performed at participant level and no account was taken of the hierarchical data structure (clusters).

The two study groups were compared using ANCOVA, with outcome variables at follow-up set as the dependent variable [47]. Baseline values were used as a covariate to control for imbalance between control and intervention groups at baseline [48]. The difference in the follow-up adjusted mean (intervention minus control) and 95% CI, are presented for each outcome measure. Standardized mean-difference effect size was calculated by using the means of the two groups, mean-square error and the correlation between the covariate and dependent variable as presented in Lipsey & Wilson [49]. Effect sizes greater than 0.8 were considered large; 0.5 was medium; and 0.2 was small [50]. Qualitative interviews were transcribed for thematic analysis employing constant comparison methodology. Descriptive and explanatory categories were thematised with representative quotes to illustrate themes [51].

## Results

### Schools characteristics and participant recruitment

All schools were based in urban areas. Table 1 presents information describing intervention and control schools in terms of: school size, deprivation, number of students in Year 7, number of students recruited and information from eligible schools that did not agree to participate. Four out of the five intervention schools provided 2 hours of physical education (PE) lessons per week to year 7 pupils, while one school provided 1 hour and 40 minutes per week. Both control schools provided 2 hours of physical education lessons per week and no further physical activity interventions were happening at these schools during the period of the study. The results also show that intervention schools that took part in the study tended to have fewer students and be more deprived than eligible schools that did not take part. The reverse was seen for control schools.

**Figure 1:**
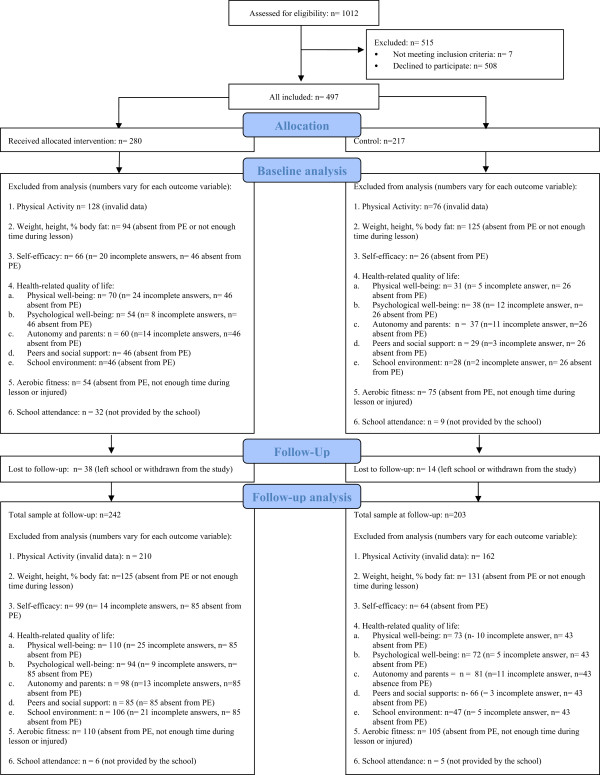
**Flowchart of participants during the dance mat exergaming intervention.**

A CONSORT-style diagram of participant flow through the study is presented in Figure 1. Although the intervention was delivered to all children in the school, only a subset of 280 participants agreed to participate in the study, and of those, 86% were measured at follow-up. In the control group 217 participants were recruited and 93% were measured at follow-up.

**Table 1:** **Characteristics of intervention and control schools**

Schools	Pupils on roll 2011	Index of multiple deprivation rank (1 = most deprived)	Free school meals eligibility (%)	Number of children recruited (number children invited)
Intervention 1	1,060	5,376	21.8	87 (185)
Intervention 2	375	5,376	48.0	20 (32)
Intervention 3	792	1,464	52.8	95 (180)
Intervention 4	1,174	20,324	51.6	37 (215)
Intervention 5	685	1,540	29.6	41 (110)
Mean (range) intervention schools	817 (375 to 1,174)	6,816 (1,464 to 20,324)	40.8 (21.8 to 52.8)	
Mean (range) eligible intervention schools not recruited	1059 (330 to 1576)	11,997 (30 to 31,728)	26.6 (10.1 to 59.5)	
Control 1	766	5,100	39.9	47 (60)
Control 2	1,224	29,926	15.4	170 (230)
Mean (range) control schools	995 (766 to 1,224)	17,513 (5,100 to 29,926)	27.7 (15.4 to 39.9)	
Mean (range) eligible control schools not recruited	815 (476 to 1,208)	7,110 (976 to 17,046)	41.0 (11.2 to 58.8)

### Descriptive data

Data were collected on at least one outcome variable at baseline from 497 participants (intervention n = 280; control n = 217). Participants’ mean age at baseline was: intervention = 11.2 ± 0.4 year; control = 11.3 ± 0.4 years (p =0.007). The gender distribution of participants was similar at baseline between study arms (intervention group 63.9% female, control group 64.5% female, p = 0.892). There was a significant difference between groups at baseline in terms of: self-efficacy for physical activity (p = 0.009), physical well-being (p = 0.015), 20 metre shuttle-run test velocity (p = 0.009), number of shuttles completed (p = 0.004), sedentary time (p < 0.001), MVPA (p = 0.003), total physical activity vertical axis (p < 0.001), total physical activity vertical magnitude (p < 0.001) and school attendance (p = 0.04). Table 2 describes information on intervention and control groups data at baseline, follow-up and difference between baseline and follow-up.

**Table 2:** **Participants’ demographic and clinical characteristics at baseline**

	Intervention			Control		
	Baseline mean ± SD (n)	Follow-up mean ± SD (n)	Difference between follow-up and baseline	Baseline mean ± SD (n)	Follow-up mean ± SD (n)	Difference between follow-up and baseline
Sedentary time (min.d^-1^)	502.3 ± 66.5 (152)	512.7 ± 63.5 (32)	28.7 ± 69.3 (28)	601.3 ± 146.0 (141)	622.2 ± 144.3 (41)	-12.2 ± 70.2 (41)
Sedentary time (% of wear time)	60.7 ± 6.3 (152)	64.9 ± 7.0 (32)	5.8 ± 6.0 (28)	62.9 ± 6.2 (141)	63.9 ± 6.4 (41)	1.8 ± 4.9 (41)
Light physical activity (min.d^-1^)	236.7 ± 44.1 (152)	211.2 ± 41.0 (32)	-46.3 ± 43.4 (28)	240.3 ± 47.3 (141)	232.4 ± 46.9 (41)	-13.9 ± 39.0 (41)
Light physical activity (% of wear time)	31.2 ± 5.0 (152)	27.8 ± 5.1 (32)	-4.8 ± 5.2 (28)	30.1 ± 4.9 (141)	28.9 ± 5.0 (41)	-1.6 ± 4.4 (41)
Moderate to vigorous physical activity (min.d^-1^)	61.3 ± 23.8 (152)	55.9 ± 24.9 (32)	-9.3 ± 17.3 (28)	55.7 ± 22.0 (141)	57.8 ± 22.2 (41)	-3.4 ± 16.5 (41)
Moderate to vigorous physical activity (% of wear time)	8.1 ± 3.2 (152)	7.3 ± 2.9 (32)	-1.0 ± 2.1 (28)	6.9 ± 2.8 (141)	7.2 ± 2.7 (41)	0.2 ± 2.1 (41)
VA total activity (counts min^-1^) (% of wear time)	528.5 ± 171.2 (152)	475.5 ± 147.6 (32)	-89.4 ± 110.6 (28)	483.5 ± 143.5 (141)	493.5 ± 200.2 (41)	-23.7 ± 129.0 (41)
VM total activity (counts min^-1^) (%)	1074.6 ± 267.8 (152)	942.1 ± 261.3 (32)	-186.1 ± 218.7 (28)	990.6 ± 237.5 (141)	976.7 ± 267.3 (41)	-72.5 ± 175.5 (41)
Weight (kg)	45.0 ± 11.8 (186)	52.7 ± 13.4 (117)	7.0 ± 4.5 (117)	43.4 ± 9.1 (92)	52.5 ± 10.4 (72)	8.7 ± 3.9 (72)
Body mass index (kg.m ^-2^)	20.4 ± 4.2 (186)	21.5 ± 4.4 (117)	0.9 ± 1.5 (117)	19.5 ± 3.3 (92)	21.3 ± 3.7 (72)	1.8 ± 1.4 (72)
Percentage body fat	23.2 ± 10.3 (186)	23.5 ± 10.9 (117)	0.1 ± 7.1 (117)	21.3 ± 8.7 (92)	23.7 ± 9.8 (72)	2.8 ± 6.5 (72)
Self- efficacy for physical activity	24.3 ± 4.5 (214)	22.8 ± 4.9 (143)	-1.1 ± 5.8 (143)	25.5 ± 4.5 (191)	22.5 ± 5.9 (139)	-3.1 ± 6.0 (139)
Physical well-being	49.3 ± 9.3 (210)	48.1 ± 8.7 (132)	-1.2 ± 10.0 (132)	51.8 ± 10.8 (186)	46.8 ± 8.2 (130)	-4.9 ± 11.7 (130)
Psychological well-being	50.9 ± 10.9 (226)	50.4 ± 10.7 (148)	0.6 ± 12.7 (148)	52.9 ± 9.1 (179)	48.8 ± 9.3 (131)	-4.1 ± 11.0 (131)
Autonomy and parent relation	52.9 ± 11.8 (220)	54.6 ± 13.2 (144)	3.4 ± 14.3 (144)	54.8 ± 11.1 (180)	52.0 ± 10.9 (122)	-2.5 ± 11.7 (122)
Peers and social support	56.2 ± 10.6 (234)	52.8 ± 11.5 (157)	-2.2 ± 12.4 (157)	54.7 ± 10.6 (188)	51.4 ± 11.0 (137)	-2.5 ± 11.6 (137)
School environment	56.1 ± 11.1 (234)	50.3 ± 10.1 (136)	-6.2 ± 13.0 (156)	55.8 ± 9.8 (189)	48.0 ± 10.1 (156)	-7.6 ± 11.3 (136)
20-m shuttle run test (km.h^-1^)	10.0 ± 0.9 (226)	10.1 ± 0.9 (132)	-0.1 ± 12.9 (132)	10.3 ± 1.0 (142)	10.3 ± 1.1 (98)	-0.3 ± 10.8 (98)
Number of shuttles completed	28.7 ± 16.8 (226)	30.2 ± 16.2 (132)	-0.03 ± 0.7 (132)	34.0 ± 18.0 (142)	34.7 ± 19.8 (98)	-0.04 ± 0.6 (98)
School attendance (%)	95.6 ± 8.0 (248)	94.8 ± 6.3 (242)	-0.8 ± 8.2 (242)	94.2 ± 5.9 (208)	93.9 ± 7.2 (203)	-0.2 ± 6.5 (203)

Sample sizes of each variable are presented on brackets.

### Use of the dance mats - qualitative data

A service level agreement with the funders of the initiative demanded that all schools participated in the initial 6 week curriculum timetabling of dance mats, after this period, use became increasingly patchy within and across schools. Barriers to the use of the mats included a decline in leadership of the programme, different expectations and values about the mats, a lack of innovation in use of mats in local settings (e.g. repetition of programmes or songs leading to boredom of students), differences in the availability of time and space, and an overall decline in funding and administrative support for the programme (including job cuts for PE teachers), a politically driven shift in focus to competitive school sports associated with a change in government and the build-up to the Olympics. Facilitators included adoption by several key ‘champions’, extension and adaptation of mats within settings, and use of the mats with pupils who were considered ‘hard to reach’ in terms of physical activity.

Table 3 provides information on the use of dance mats in schools and shows substantial variation in the use of the dance mats between schools with use during lunchtimes, after school and during the physical education curriculum.

**Table 3:** **Use of dance mats in schools**

School	Use of dance mats
Intervention 1	Incorporated into 8 week ‘blocks’ of physical education lessons during the winter months and occasional use in PE classes at other times if inclement weather. Also used during lunch times for a ‘dance club’ but with teachers noting ‘drop off’ in interest over time.
Intervention 2	Used in PE lessons between February and March 2011. Teachers reported early interest with drop off in engagement after ‘a few weeks’.
	Available at lunch and morning break time throughout the year
Intervention 3	Incorporated in PE lessons for fitness classes in 6 week ‘blocks’.
	Provided during enrichment lessons after school.
	Occasionally available at lunch time.
Intervention 4	Not used consistently during curriculum time, however two teachers reported use in fitness programme at the beginning of the programme for several weeks. Available at lunch time, during inclement weather for sports classes, and for an organised lunch time ‘dance mat club’
Intervention 5	Intermittently used during PE lessons if suitable space available (e.g. during exam period).

The findings of the qualitative work overall suggest that contextual issues in the introduction of the dance mats moderated the overall intensity of effects downwards because of declining support for the initiative, but with several local exceptions where there were higher levels of use by some pupils and in some settings.

#### Analysis of change over time

Table 4 shows the actual and adjusted follow-up means and difference in adjusted means for all outcome variables in intervention and control participants.

**Table 4:** **Follow-up adjusted for baseline covariates and mean difference (intervention-control)**

	Intervention	Control	Mean difference (intervention minus control) (95% CI)	p value	Effect size (95% CI)
Sedentary time (min.d^-1^)	503.9 ± 64.2	507.2 ± 63.4	-3.3 (-35.3 to 28.6)	0.83	-0.05 (-0.53 to 0.43)
Sedentary time (% of wear time)	66.5 ± 5.2	63.1 ± 5.2	3.3 (0.7 to 5.9)	0.01*	0.54 (0.05 to 1.03)
Light physical activity (min.d^-1^)	205.6 ± 36.0	234.3 ± 36.4	-28.7 (-46.5 to -10.8)	0.02*	-0.68 (-1.17 to -0.18)
Light physical activity (% of wear time)	27.0 ± 4.4	29.3 ± 4.3	-2.3 (-4.5 to 0.2)	0.03*	-0.46 (-0.94 to 0.03)
MVPA (min.d^-1^)	52.2 ± 16.4	58.2 ± 16.0	-5.6 (-13.6 to 2.3)	0.16	-0.26 (-0.74 to 0.22)
MVPA (% of wear time)	6.7 ± 2.1	7.4 ± 2.1	-0.6 (-1.6 to 0.4)	0.21	-0.03 (-0.51 to 0.45)
VA total activity (counts min^-1^)	442.1 ± 123.3	507.5 ± 122.9	-65.4 (-126.0 to -4.7)	0.03*	-0.37 (-0.85 to 0.12)
VM total activity (counts min^-1^)	892.5 ± 187.2	993.0 ± 230.7	-100.5 (-193.3 to -7.6)	0.03*	-0.39 (-0.89 to 0.09)
Weight (kg)	52.0 ± 4.3	53.7 ± 4.3	-1.7 (-2.9 to -0.4)	0.01*	-0.14 (-0.43 to 0.16)
Body mass index (kg.m ^-2^)	21.1 ± 1.5	21.9 ± 1.5	- 0.9 (-1.3 to -0.4)	0.0001*	-0.21 (-0.51 to 0.082)
Percentage body fat	22.8 ± 6.7	25.0 ± 6.7	-2.2 (-4.2 to -0.2)	0.03*	-0.20 (-0.49 to 0.09)
Self-efficacy for physical activity	23.1 ± 5.2	22.2 ± 5.2	0.9 (-0.3 to 2.2)	0.13	0.17 (-0.06 to 0.41)
Physical well-being	48.4 ± 8.2	46.5 ± 8.2	1.9 (0.2 to 4.8)	0.06	0.23 (-0.02 to 0.47)
Psychological well-being	50.8 ± 9.7	48.3 ± 9.7	2.5 (0.1 to 4.8)	0.03*	0.25 (0.01 to 0.48)
Autonomy and parent relation	55.5 ± 11.4	51.3 ± 11.4	4.2 (1.4 to 7.0)	0.003*	0.34 (0.10 to 0.59)
Peers and social support	52.6 ± 10.3	51.7 ± 10.3	0.9 (-1.5 to 3.3)	0.45	0.08 (-0.15 to 0.31)
School environment	50.2 ± 9.9	48.2 ± 9.9	2.0 (-0.2 to 4.3)	0.09	0.19 (-0.04 to 0.42)
20 m shuttle run test (km.h^-1^)	10.2 ± 0.7	10.2 ± 0.7	0.0 (-0.2 to 0.1)	0.72	-0.03 (-0.29 to 0.23)
Number of shuttles completed	31.8 ± 11.6	32.6 ± 11.6	-0.8 (-3.8 to 2.3)	0.61	-0.04 (-0.30 to 0.21)
School attendance (%)	94.6 ± 6.2	94.2 ± 6.2	0.3 (-0.8 to 1.4)	0.57	0.05 (-0.14 to 0.24)

*Significantly different between groups. MVPA – moderate to vigorous physical activity; VA - vertical axis; VM - vertical magnitude.

There was good compliance with accelerometers at baseline with 54.2% (n = 152) of intervention and 64.9% (n = 141) of control group participants meeting the minimum wear-time requirements for inclusion in the analysis. However, compliance dropped significantly at follow-up with only 11% (n = 32) of the intervention and 19% (n = 41) of control group participants meeting the wear-time requirements. The average number of valid days and hours at baseline and follow-up were:

A. Baseline: intervention 4.8 ± 1.4 days and 12.6 ± 1.0 hours; and control 4.9 ± 1.1 and 13.2 ± 1.1 hours.
B. Follow-up: intervention 4.5 ± 1.4 days and 12.6 ± 1.0 hours; and control 4.6 ± 1.1 and control 13.4 ± 1.2.

There was a significant difference in adjusted means between intervention and control groups for: percentage of sedentary time (mean difference = 3.3%, 95% CI = - 0.7 to -5.9, p = 0.01); light physical activity (mean difference = -28.7, 95% CI = -46.5 to -10.8, p = 0.02), percentage of light physical activity (mean difference = -2.3%, 95%CI = -4.5 to 0.2, p = 003), and VA and VM total physical activity (mean difference VA total physical activity = -65.4 cpm, 95% CI = -126.0 to -4.7, p = 0.03 and mean difference VM total physical activity = -100.5 cpm, 95% CI = --193.3 to -7.6, p = 0.03).

There was a significant difference between intervention and control groups adjusted means at follow-up for body weight (mean difference = -1.7 kg, 95% CI = -2.9 to -0.4, p = 0.01), BMI (mean difference = -0.9 kg/m^2^, 95% CI = -1.3 to -0.4, p = 0.0001) and body fat percentage (mean difference = - 2.2%, 95% CI = -4.2 to - 0.2, p = 0.03).

There was no statistical difference between intervention and control participants between follow-up adjusted means for self-efficacy for physical activity or aerobic fitness. However, there was a significant difference between intervention and control groups adjusted means for psychological well-being and autonomy and parent relation in the intervention group compared to control group. There were no statistical differences between groups for physical well-being, peers and social support and school environment. There was no difference in school attendance between the two groups.

## Discussion

### Summary of results

This is the first large, pragmatic trial of exergaming in schools that we are aware of. There was limited evidence that the intervention was associated with harmful changes in some physical activity parameters but this result should be interpreted with care as there was very poor compliance with accelerometers at follow-up. However, implementation of the dance mat intervention was associated with improvements in weight, BMI, percentage of body fat and some dimensions of health-related quality of life: psychological well-being, and autonomy and parent relation. There was no evidence that implementation of the intervention was associated with changes in aerobic fitness, school attendance, or three other dimensions of health-related quality of life: physical well-being, peer and social support, and school environment. The results from the qualitative analysis, while only briefly described here, demonstrate some elements of the complex political and social setting(s) into which the dance mats were placed. The qualitative findings suggest that potential benefits of the dance mat technology have been under rather than over-estimated in the measurement of outcomes at the population level.

### Interpretation of results

It has been reported that dance mat exergame can elicit moderate to vigorous physical activity [52]. However, in this study MVPA was not significantly different between groups. Although this was an unexpected result, it reflects other controlled trial with large sample size which has not found a significant effect of exergaming on MVPA [6]. Likewise, there was an unexpected significant and clinically relevant (medium effect size) decrease on light physical activity (min/day and percentage) and an increase in percentage of sedentary time on intervention group compared to control group. This was directly related to the decrease in total physical activity (of around 10%) seen in this group compared to an increase (of around 3%) seen in the control group. The decrease in physical activity in the intervention group is high compared to the drop reported from a cross-sectional study of 4% a year seen in girls [27]. This unanticipated result could have been due to other changes in intervention schools not associated with the intervention which affected physical activity (e.g. increase in study time). However, we are not aware of any such changes and none were identified in the qualitative data.

The negative results in relation to physical activity found in this study are difficult to interpret particularly when considered alongside the positive changes in body composition associated with the intervention. However, this result needs to be interpreted with caution as there were limited data on physical activity at follow-up due to low compliance with accelerometers. This is an important limitation of the study. Possible reasons for low compliance at follow-up include: loss of interest from children (accelerometers were no longer a novelty); and the long gap between baseline and follow-up measurements (approximately 12 months). Participants were always reminded of the importance of wearing the accelerometer at both data collection points, but this may not have been reinforced by parents and teachers. Participants also received a £10 voucher when the accelerometer was returned at the end of the study, which reduced the loss of the accelerometers at follow-up compared to baseline. However, extra incentives could have been provided if accelerometers were worn for the required period (3 days – 10 hours). Unfortunately, the use of accelerometers was not explored in the qualitative study as we did not anticipate that this would be an issue when data were collected.

The improvement in body composition associated with implementation of the intervention reflects similar findings from a large randomised controlled trial in which overweight and obese children were given an active video game to play at home [6]. Another multifaceted community-based pilot study (including an exergaming and exercise program, and nutrition and behavioural education) with overweight and obese children also found a significant improvement in BMI after 10 weeks [53]. However, the results of the present study were not in agreement with another smaller randomised controlled trial over 12 weeks in which children received an active video game to play at home which reported no effect on body composition [54].

The intervention effects found here in relation to BMI and percentage body fat were larger than those reported by Maddison et al., 2011 [6] (BMI: -0.9 kg/m^2^ vs. -0.25 kg/m^2^ previously; and percentage of body fat: -2.2% vs. -0.8% previously). Possible explanations for these differences include: different exposure periods (approximately 12 months in this study versus 6 months previously) and differences in the nature of the intervention (participants were given active video games to play at home rather than at school). In a follow-up study [55], the authors reported that the improvement seen in body composition was most likely to be mediated by improved aerobic fitness.

In the current study, adjusted aerobic fitness at follow-up was not significantly different between groups. Improvements in aerobic fitness associated with exergaming have been seen in some studies [12, 56, 57] but not another [6]. General information on how each school used the dance mats were recorded (Table 3). However, individual level data on use was not recorded, and the absence of effect here may be due to differences in intensity, duration and frequency of use compared to previously conducted studies.

Results from the Kidscreen-27 data revealed that the intervention was associated with beneficial effects in psychological well-being and autonomy and parent relation. The psychological well-being dimension explores positive emotions and satisfaction with life, whilst the autonomy and parent relation dimension focuses on the quality of interaction and support between the child and parent or carer and the perceived level of autonomy and financial resources available to the child [43].

Exergaming has previously been reported to be associated with a greater drop in positive well-being from pre- to post-exercise than treadmill exercise [58], but long term psychological effects of exergaming have not been investigated. Previous studies have reported that health-related quality of life decreases in most parameters across adolescence [59, 60]. The positive results from the present study indicate that exergaming may be an effective strategy to maintain, or improve, some health-related quality of life parameters in adolescence.

There was no relationship between implementation of the dance mat intervention and school attendance. We are not aware of any previous research on school attendance and exergaming.

The intervention was applied in an unsettled political period with national changes to the management and structure of both primary care and school sports. Along with a loss of support from the dance mat providers due to bankruptcy, this effected the support schools received during the implementation and delivery period with impacts on training, equipment maintenance and support to embed the dance mat scheme in the school setting.

### Strengths and limitations

Although some previous studies have explored the efficacy of exergaming interventions in selected populations, as far as are aware, this is the first natural experiment exploring the effectiveness of an exergaming intervention in a ‘real-world’ setting. Furthermore, few previous studies have included the diverse range of psychosocial and physiological outcomes assessed in this study. Finally this study included a much longer follow-up (approximately 12 months) than previously (12 to 24 weeks). However, more data points with short term follow-ups (e.g. 3, 6 and 9 months) might have been revealing, as positive effects might have been seen at short term but lost at long term (12 months).

This study was based on a natural experiment, which tried to minimise bias by using schools in a neighbouring educational district as controls. However, the non-randomised design meant there were some important differences between intervention and control schools at baseline (Table 1). Although there was an attempted to match control schools to intervention schools in terms of size, deprivation and physical education provision at baseline, only two control schools were recruited and there were significant differences between both intervention and control schools and participants at baseline (Table 2). To counterbalance these differences the analysis for baseline covariates was adjusted at the individual level.

Another possible limitation was that the intervention was not underpinned by any particular theoretical framework. As noted, the intervention was developed by primary care, government and school sports leads and, as researchers, we had no input into it. Although it has been proposed that physical activity interventions should be based on theoretical frameworks [61], there is limited evidence that this is advantageous for community interventions [62].

We do not have full data on all outcome variables for all children in the sample (information about sample sizes for each measurement are shown in brackets in Tables 2 and 4 for baseline and follow-up respectively). As data were collected during lesson time, there were substantial restrictions in the time available for data collection and children were sometimes absent from school.

Although we have investigated some components of the process evaluation in the qualitative study (e.g. how the intervention was implemented and contextual factors that might have affected the intervention), a detailed process evaluation was not conducted. In particular, we did not perform a detailed analysis of fidelity, dose or programme content.

### Implications for policy, practice and research

Given the wide variation in how the intervention was implemented between schools (Table 3), further work is required to establish the most effective approaches to use. In particular, restricting exergaming to scheduled physical education lessons may not be the most effective approach to implementation, as it merely substitutes one form of physical activity for another. Further strategies need to be developed for making use of the intervention during periods that children are likely to be otherwise inactive (e.g. lunch time, breaks and after school).

Although there was no improvement in physical activity associated with the intervention, there was an improvement in children’s body composition - a finding supported by other studies [6, 53]. This suggests that exergaming may be particularly effective as a weight reduction or weight control intervention and this should be investigated further.

Furthermore, the novel finding that the intervention was associated with improvements in some aspects of health related quality of life indicates that the benefits of exergaming in schools are wider than traditional outcomes such as physical activity. These should be explored further and communicated to wider audiences.

Finally, the dance mats were a novelty for teachers and children during the 12 months of the intervention. However, the attractiveness of this innovative intervention may not be sustainable over the longer term. Longer term effects, and methods of maximising these require further investigation.

## Conclusions

The implementation of a dance mat exergaming scheme in public secondary schools was associated with improvement in weight, BMI, percentage of body fat and some parameters of health-related quality of life, but not aerobic fitness, self-efficacy for physical activity, or school attendance. The contextual issues in which dance mats was implemented in schools suggested that the potential benefits might have been under-estimated. However, these results need to be interpreted with caution as there were insufficient data on physical activity at follow up to draw robust conclusions.
